# The complete mitochondrial genome and phylogenetic analysis of scyphozoan jellyfish *Chrysaora chinensis* (Cnidaria: Scyphozoa: Pelagiidae) in the coastal sea of Fangchenggang, China

**DOI:** 10.1080/23802359.2026.2627024

**Published:** 2026-02-17

**Authors:** Tong Su, Jie Guo, Yutong Xie, Bingfu Tan, Mingben Xu, Liangliang Huang, Junxiang Lai

**Affiliations:** a Guangxi Key Laboratory of Marine Environmental Science, Guangxi Academy of Marine Sciences, Guangxi Academy of Sciences, Nanning, China; b Guangxi Key Laboratory of Theory and Technology for Environmental Pollution Control, Guilin University of Technology, Guilin, China

**Keywords:** *Chrysaora chinensis*, scyphozoa, mitogenome, phylogenetic analysis

## Abstract

The scyphozoan jellyfish *Chrysaora chinensis* has been frequently observed in the coastal waters of Fangchenggang, China, in recent years. This study assembled and analyzed the first complete mitochondrial genome (mitogenome) of *C. chinensis* from next-generation sequencing data. The mitogenome was 16,783 bp in length, exhibited an overall AT content of 69%, and encoded 13 typical protein-coding genes (PCGs), 2 ribosomal RNA (rRNA) genes (*rrnS* and *rrnL*), 2 transfer RNA (tRNA) genes (*trnW-tca* and *trnM-cat*), and an additional open reading frame (ORF), *dpo*. The mitochondrial gene order was largely consistent with that of previously reported jellyfish species. Furthermore, phylogenetic analysis of 13 mitochondrial PCGs from 19 jellyfish species indicated that *Chrysaora chinensis* is closely related to *Chrysaora pacifica* and *Chrysaora quinquecirrha*. These results provided a valuable reference for clarifying the taxonomy, evolutionary relationships, and phylogeography of scyphozoan jellyfish.

## 1. Introduction

In recent years, jellyfish blooms have become increasingly frequent in coastal waters worldwide (Dong et al. 2010; Kennerley et al. 2022; Suárez et al. 2022). Such blooms repeatedly damage marine ecosystems and coastal economies, and also endanger human health and safety (Purcell et al. 2007; Wang et al. 2023; Osathanunkul, 2024). The scyphozoan jellyfish *Chrysaora chinensis* Vanhöffen, 1888 (Cnidaria: Scyphozoa: Semaeostomeae: Pelagiidae), first discovered in the South China Sea near Hong Kong, is named for its type locality (Morandini and Marques, 2010). It is widely distributed in the Western Pacific Ocean and has been observed off the coasts of Indonesia, Sumatra, China, and the Philippines (Morandini and Marques, 2010). Notably, this species has been frequently observed in the waters near Fangchenggang, Qinzhou Bay, China, over the past two years. The jellyfish within the genus *Chrysaora*, commonly referred to as sea nettles, are characterized by umbrella-shaped bells that can reach several tens of centimeters in diameter, as well as numerous elongated tentacles equipped with nematocysts. They exhibit considerable adaptability to a range of water temperatures and salinities (Morandini and Marques, 2010). The safety of the cold source water intake at Fangchenggang Nuclear Power Plant may be compromised by large aggregations of *C. chinensis*. However, studies on *C. chinensis* have been primarily morphology-focused, with mitochondrial gene sequencing efforts concentrated on specific genes such as *cox1* and 16S rRNA (Daglio and Dawson, 2017; Low et al. 2019).

At the class level, database and literature surveys combined show that only 29 scyphozoan mitogenomes—assigned to 22 nominal species (20 complete, 2 partial)—have been published to date (“Scyphozoa[Organism] AND mitochondrion[Title]”, NCBI, accessed 6 January 2026). As of now, the NCBI Nucleotide database contains two complete mitogenomes of *Chrysaora chinensis*: PV929673 (this study, assembled and released by us on 20 July 2025) and a later entry (PX261967). We used the first-available mitogenome of *C. chinensis* to reconstruct its phylogeny and provide genomic resources for *Chrysaora* systematics and broader insights into jellyfish evolution.

## 2. Materials and methods

**Figure 1.**
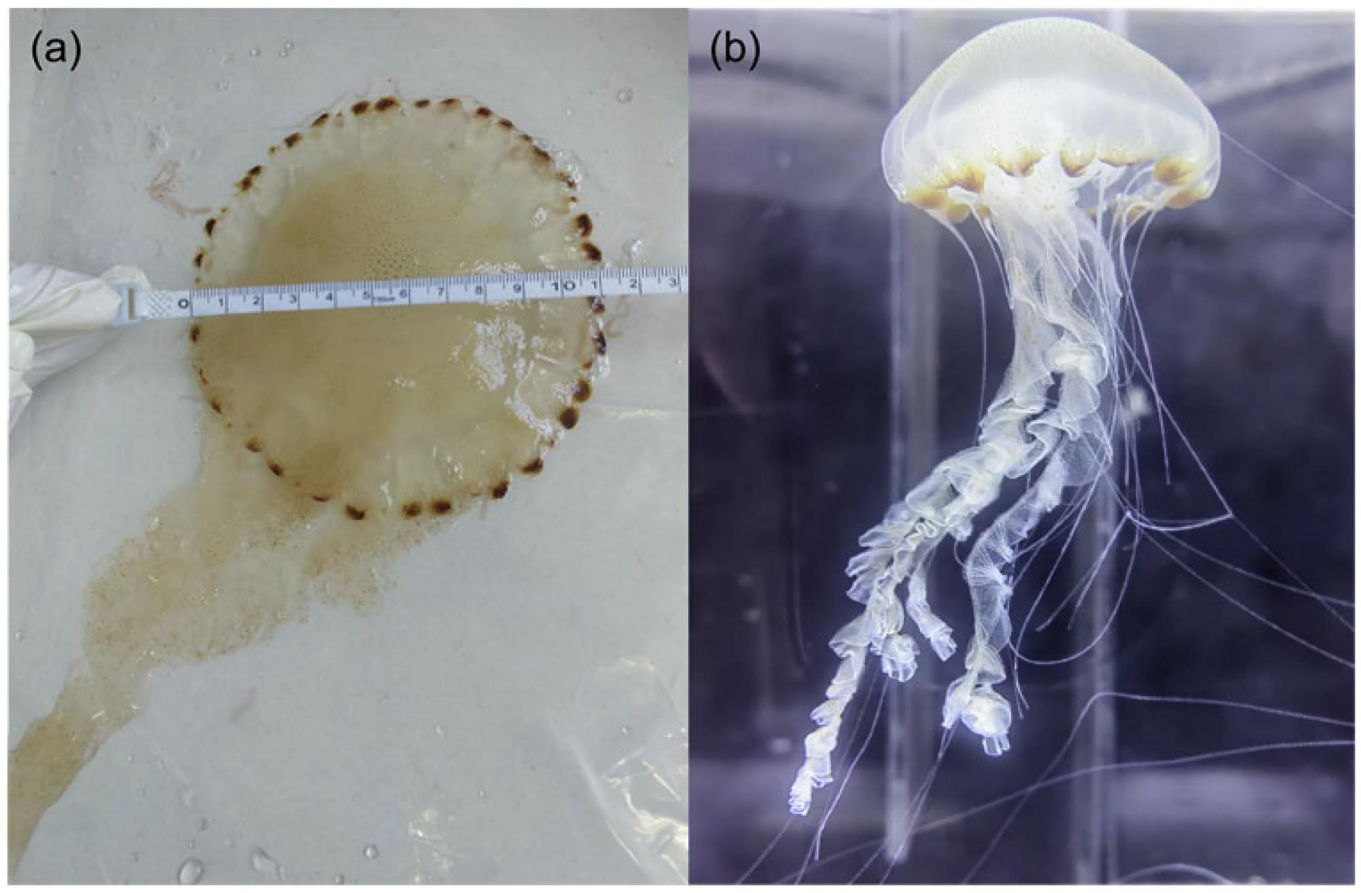
Reference images of *Chrysaora chinensis*. (a) Close-up view of the umbrella (diameter approximately 11 cm) and oral arms (length approximately 26 cm). (b) Overall view of the jellyfish. These photographs were taken by the author Tong Su in December 2024 at the Laboratory of the Guangxi Academy of Marine Sciences, China.

*Chrysaora chinensis* was collected from the coastal waters of Fangchenggang (108°36′37.79″E, 21°37′51.96″N) in Qinzhou Bay, China, using a plankton net on 25 December 2024. The specimen was deposited at the Laboratory of Marine Environmental Science, Guangxi Academy of Sciences (Dr. Jie Guo, guojie2575@163.com) under voucher number *Chrysaora*2024-1201 (Figure 1). Species identity was verified by combining morphological analysis (Morandini and Marques, 2010; Low et al. 2019) and sequencing of the commonly used mitochondrial markers *cox1* and 16S rRNA; the resulting sequences showed a high degree of similarity to GenBank accessions KY611280 and MF141690, respectively. From *C. chinensis* umbrella muscle, genomic DNA was extracted using the DNeasy Tissue Kit (Qiagen, Beijing, China). Prior to NovaSeq library construction, 1 µg of high-quality DNA was sheared to 300–500 bp using a Covaris M220 focused-ultrasonicator; short-insert libraries were then prepared and sequenced on the NovaSeq 6000 platform (BIOZERON Co., Ltd., Shanghai, China), yielding 150 bp paired-end reads. GetOrganelle v1.7.5 was used to assemble the mitogenome (Jin et al. 2020). Assembly completeness and coverage uniformity were assessed by read-depth profiling (Supplementary Figure S1). Mitogenome genes were annotated with MITOS2 (Bernt et al. 2013), and tRNA genes were predicted by tRNAscan-SE (Chan and Lowe, 2019). The complete mitogenome map was then visualized using CGView (Stothard et al. 2019).

A molecular phylogenetic reconstruction of the class Scyphozoa was performed using 19 mitochondrial genomes, comprising 17 scyphozoan species and 2 hydrozoan outgroups (*Eirene ceylonensis* and *Craspedacusta sowerbii*) specifically selected to enhance phylogenetic resolution (Table 1). The mitogenomes were retrieved from NCBI GenBank, including the newly sequenced mitogenome of *C. chinensis* generated in this study. The phylogeny was inferred from a concatenated nucleotide supermatrix of the 13 mitochondrial PCGs shared by most jellyfish species. These sequences were processed using Phylosuite v1.2.3, which included performing multiple sequence alignment with MAFFT, trimming ambiguous regions and individual columns exhibiting excessive gaps or ambiguities using trimAI and Gblocks, followed by concatenating the cleaned alignments (Zhang et al. 2020). A maximum-likelihood (ML) phylogenetic tree was constructed in MEGA 12 with 1,000 bootstrap replicates (Kumar et al. 2024), and subsequently visualized and manually refined using iTOL (https://itol.embl.de/).

**Table 1.** Nineteen jellyfish mitogenomes for phylogenetic analysis.

Species name	Accession number	Length	Order	Reference
*Chrysaora chinensis*	PV929673	16,783 bp	Semaeostomeae	This study
*Chrysaora pacifica*	MN448506	16,964 bp	Semaeostomeae	(Wang and Yin, 2020)
*Chrysaora quinquecirrha*	MW401676	16,875 bp	Semaeostomeae	unpublished
*Pelagia noctiluca*	OQ446325	16,390 bp	Semaeostomeae	(Lee and Ki, 2023)
*Cyanea capillata*	JN700937	16,202 bp	Semaeostomeae	(Kayal et al. 2012)
*Aurelia aurita*	HQ694729	16,971 bp	Semaeostomeae	(Park et al. 2012)
*Aurelia coerulea*	MT023105	16,748 bp	Semaeostomeae	(Seo et al. 2020)
*Aurelia limbate*	MK527107	16,953 bp	Semaeostomeae	(Karagozlu et al. 2019)
*Nemopilema nomurai*	KY454767	17,024 bp	Rhizostomeae	(Wang and Sun, 2017a)
*Rhopilema esculentum*	KY454768	15,855 bp	Rhizostomeae	(Wang and Sun, 2017b)
*Acromitus flagellatus*	OM457248	16,779 bp	Rhizostomeae	(Lin et al. 2022)
*Catostylus townsendi*	OK299144	16,308 bp	Rhizostomeae	unpublished
*Cassiopea xamachana*	JN700936	15,949 bp	Rhizostomeae	(Kayal et al. 2012)
*Cassiopea andromeda*	JN700934	15,800 bp	Rhizostomeae	(Kayal et al. 2012)
*Cotylorhiza tuberculata*	OQ850984	16,590 bp	Rhizostomeae	(Jiang et al. 2024)
*Catostylus mosaicus*	JN700940	14,847 bp	Rhizostomeae	(Kayal et al. 2012)
*Mastigias papua*	OQ695499	16,560 bp	Rhizostomeae	(Xia et al. 2023)
*Craspedacusta sowerbii*	MZ508273	18,020 bp	Limnomedusae	unpublished
*Eirene ceylonensis*	OR149020	14,997 bp	Leptothecata	(Chen et al. 2023)

## 3. Results

**Figure 2.**
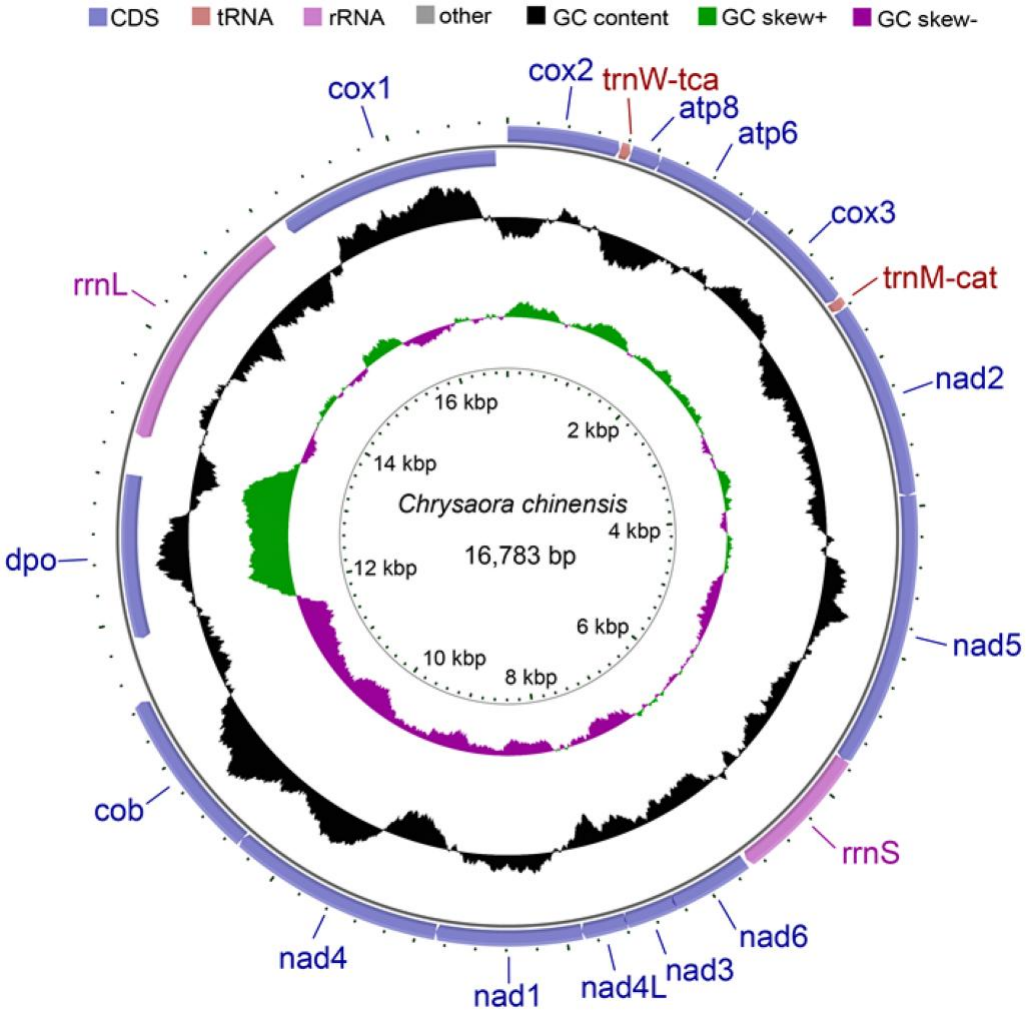
Circular mitogenome map of *Chrysaora chinensis* from Fangchenggang coastal waters.

The complete mitogenome of *C. chinensis* spanned 16,783 bp and encoded 13 typical PCGs (*cox1*, *cox2*, *atp8*, *atp6*, *cox3*, *nad2*, *nad5*, *nad6*, *nad3*, *nad4L*, *nad1*, *nad4*, *cob*), 2 rRNA genes (*rrnL* and *rrnS*), 2 tRNA genes (*trnW-tca* and *trnM-cat*) and 1 additional ORF (*dpo*) (Figure 2). Immediately downstream of *rrnL*, the 1,161-bp *dpo* was predicted to encode a 386-amino-acid protein with extensive similarity to family B DNA polymerases. All PCGs were located on the H-strand, and nearly all were in the positive direction, with the exception of *rrnL*, *cox1* and *dpo*, which were in the negative direction. Regarding the start and stop codons, the PCGs initiated with ATA (*nad3, dpo*), GTG (*nad4*), TTA (*cox1*), or ATG (all remaining genes), and terminated with TAG (*cox3*, *nad5*, *nad6, dpo*) or TAA (the rest). The mitogenome showed a pronounced AT bias, with an overall base composition of 69.00% AT (A: 31.67%, T: 37.33%) and 31.00% GC (G: 15.99%, C: 15.01%). The 13 typical PCGs occupied 11,976 bp (71.36% of the mitogenome); the predicted *rrnS* (928 bp) and *rrnL* (1,686 bp) were located between *nad5*–*nad6* and *dpo*–*cox1*, respectively.

**Figure 3.**
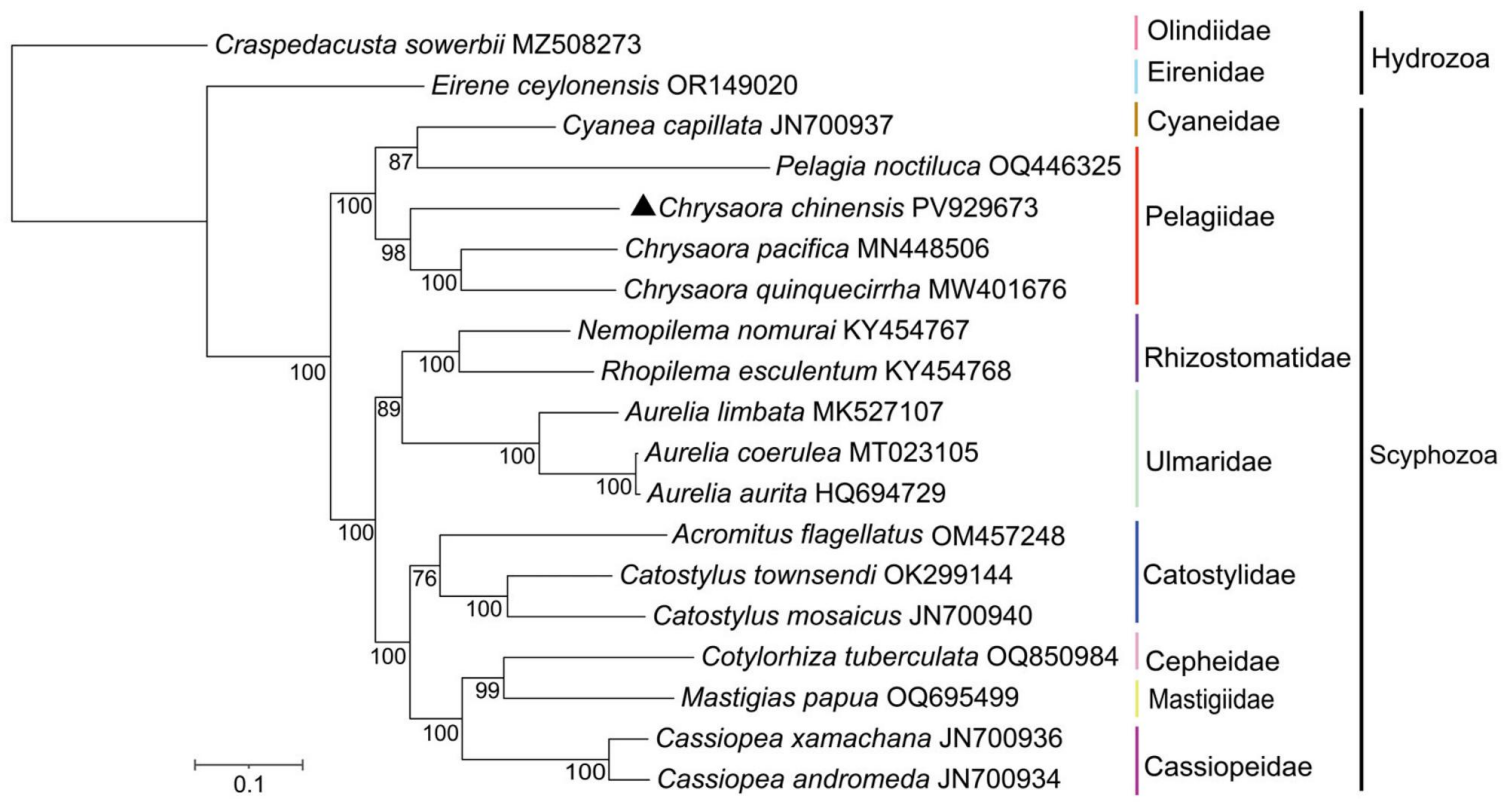
Maximum-likelihood phylogenetic tree of 19 jellyfish species based on the 13 protein-coding genes.

Before phylogenetic inference, we updated the NCBI search (6 January 2026). Subsequent retrieval of a newly deposited mitogenome (PX261967, submitted 7 weeks after ours) revealed 99.77% identity across the full length relative to our assembly, consistent with intraspecific sequence variation; it was therefore excluded from phylogenetic analyses to avoid redundancy. Maximum likelihood analysis revealed two robust clades within Scyphozoa (Figure 3). One clade included six families (Rhizostomatidae, Ulmaridae, Catostylidae, Cepheidae, Mastigiidae, and Cassiopeidae), while the other consisted of Cyaneidae and Pelagiidae. The mitogenome of *C. chinensis* was sister to the clade formed by *C. pacifica* and *C. quinquecirrha*, together constituting a distinct *Chrysaora* lineage.

## 4. Discussion and conclusions

In this study, we sequenced and characterized the complete 16,783-bp mitogenome of *Chrysaora chinensis* using high-throughput Illumina sequencing, providing the first comprehensive account of its full-length sequence, gene content and base composition. Gene order and content were highly similar to those reported for other scyphozoan jellyfish (Seo et al. 2020; Wang and Yin, 2020; Jiang et al. 2024). The distribution of genes, predominantly on the H-strand and in the positive direction except for *rrnL*, *cox1* and *dpo*, may influence the essential replication and transcription processes fundamental to mitochondrial function (Tzagoloff and Dieckmann, 2021). In this study, we predicted a 1,161-bp ORF (*dpo*) immediately downstream of *rrnL* in the *C. chinensis* mitogenome; the deduced amino-acid sequence displayed extensive similarity to family B DNA polymerases. An ORF of identical length at the syntenic position is present in the previously published mitogenome of *Craspedacusta sowerbyi* (Zou et al. 2012), and comparable ORFs have been reported in the scyphozoans *Mastigias papua* and *Cotylorhiza tuberculata* (Xia et al. 2023; Jiang et al. 2024). Collectively, the occurrence of these *dpo*-like ORFs is thought to reflect linear-plasmid integration events (Kayal et al. 2012). Hydroidolinan hydrozoans, however, appear to have lost this ORF, suggesting lineage-specific evolutionary trajectories or alternative functional compensation mechanisms that warrant further experimental investigation (Kayal et al. 2012; Ladoukakis and Zouros, 2017). Nevertheless, within Cnidaria, the absence of *dpo* may also stem from annotation practices that tend to recognize only the typical set of 13 PCGs. The high sequence variability of *dpo*-like ORFs, together with limited scrutiny of non-core regions, could lead to their being overlooked.

Consistent with most cnidarians reported to date, *C. chinensis* possessed only two 2 rRNA genes (*rrnL* and *rrnS*) and 2 tRNA genes (*trnW-tca* and *trnM-cat*), The *trnW-tca* and *trnM-cat*) were 70 bp and 71 bp, respectively, and showed high conservation across cnidarians (Kayal et al. 2012; Zou et al. 2012). The RNA genes had a slightly higher AT content than the PCGs. Medusozoan mt-tRNAs exhibited greater primary-sequence and secondary-structure variability than their anthozoan and demosponge homologues (Wang and Lavrov, 2008). The consensus secondary structures inferred for Hydrozoa, Scyphozoa and Staurozoa revealed class-specific differences in acceptor-stem length, deletions within the D-arm/D-loop region and nucleotide losses (Kayal et al. 2012). In addition, available partial cubozoan mitochondrial sequences have been reported to lack tRNA genes (Kayal et al. 2012). Accordingly, these mt-tRNA variations provide comparative reference points that merit further testing in future class-level studies of Medusozoa.

Phylogenetic analysis of 13 typical PCGs from 19 jellyfish taxa revealed that *C. chinensis* clustered with *C. pacifica* and *C. quinquecirrha*, forming a clade that was genetically distinct from other taxa (Figure 3). Differences in their PCGs among these species may be related to geographical population isolation and site polymorphisms. The need for expanded mitochondrial genome data within the Pelagiidae family was evident, given the limited information available. In conclusion, this study provided a complete mitochondrial reference for *C. chinensis*, which will facilitate future phylogenetic, phylogeographic, and evolutionary studies of scyphozoans.

## Supplementary Material

Supplemental material.docx

## Data Availability

The genome sequence data that support the findings of this study are openly available in GenBank of NCBI at (https://www.ncbi.nlm.nih.gov/) under the accession no. PV929673. The associated BioProject, BioSample, and SRA numbers are PRJNA1287265, SAMN49815718, and SRR34425592, respectively; the original sequence remains unchanged, and an annotated update, which includes additional annotation of the *dpo* gene, has been deposited in GenBase under accession C_AA126008.
