# Supplementary material for: The complete mitochondrial genome and phylogenetic analysis of scyphozoan jellyfish Chrysaora chinensis (Cnidaria: Scyphozoa: Pelagiidae) in the coastal sea of Fangchenggang, China
Source: Mitochondrial DNA B Resour. 2026 Feb 17;11(3):424–8. doi: 10.1080/23802359.2026.2627024 (PMC12915389; doi:10.1080/23802359.2026.2627024)
Supplement: Supplemental material.docx [file TMDN_A_2627024_SM6610.docx]

**Supplementary Material**

**

The complete mitochondrial genome and phylogenetic analysis of scyphozoan jellyfish** ***Chrysaora chinensis* (Cnidaria:** **Scyphozoa: Pelagiidae) in the coastal sea of Fangchenggang, China**

Figure S1. Coverage depth plot of the *Chrysaora chinensis* mitochondrial genome. The x-axis represents the nucleotide position, and the y-axis represents the coverage depth.
